# Supplementary material for: Genome-Wide Association Study of Potential Meat Quality Trait Loci in Ducks
Source: Genes (Basel). 2022 May 31;13(6):986. doi: 10.3390/genes13060986 (PMC9222319; doi:10.3390/genes13060986)
Supplement: Supplementary file 1 [file genes-13-00986-s001.zip › genes-1662673-supplementary.pdf]

genes

Table S1. Information of all significant SNP of all meat quality trait.

Phenotype	SNP	Chromosome	Position	P value	Gene	Location
Fat	SNP11963088	8	31899323	2.97E-07	BARHL2	intergenic region
	SNP11963100	8	31903087	6.55E-07		intergenic region
	SNP11963122	8	31908362	9.20E-07		intergenic region
	SNP11963132	8	31909389	1.11E-07		intergenic region
	SNP11963134	8	31910013	2.52E-08		intergenic region
	SNP11963135	8	31910023	1.54E-07		intergenic region
	SNP11963148	8	31913227	1.53E-07		intergenic region
	SNP11963190	8	31921556	3.04E-07		intergenic region
	SNP11963192	8	31923107	5.54E-07		intergenic region
	SNP11963213	8	31927164	1.26E-07		intergenic region
	SNP11963220	8	31928336	5.86E-07		intergenic region
	SNP11963251	8	31935878	4.12E-07		intergenic region
	SNP11963252	8	31935960	1.46E-07		intergenic region
	SNP11963257	8	31936298	9.62E-07		intergenic region
	SNP11963273	8	31939460	2.84E-07		intergenic region
	SNP11963278	8	31941440	3.48E-07		intergenic region
	SNP11963280	8	31941563	5.06E-07		intergenic region
	SNP11963297	8	31945764	2.57E-07		intergenic region
	SNP11963301	8	31947006	7.06E-07		intergenic region
	SNP11963327	8	31955461	8.36E-07		intergenic region
	SNP11963328	8	31955575	2.79E-07		intergenic region
	SNP11963338	8	31957707	1.91E-07		intergenic region
	SNP11963364	8	31967403	9.40E-07		intergenic region
	SNP11963365	8	31969582	1.07E-07		intergenic region
	SNP11963371	8	31969958	1.34E-07		intergenic region
	SNP11963407	8	31976272	2.13E-07		intergenic region
	SNP11963415	8	31979906	5.52E-08		intergenic region
	SNP11963439	8	31981466	6.26E-07		intergenic region
	SNP11963440	8	31982450	1.77E-07		intergenic region
	SNP11963444	8	31982878	6.27E-07		intergenic region
	SNP11963447	8	31983327	8.95E-07		intergenic region
	SNP11963457	8	31985964	4.63E-07		intergenic region
	SNP11963467	8	31990934	1.33E-07		intergenic region
	SNP11963468	8	31990944	3.19E-07		intergenic region
	SNP11963479	8	31992271	4.37E-08		intergenic region
	SNP11963534	8	32004868	7.13E-07		intergenic region
	SNP11963928	8	32105050	3.95E-07		intergenic region
	SNP11964029	8	32146498	6.21E-07		intergenic region
	SNP2012689	10	983671	6.63E-07	COPS7B	intergenic region
	SNP2012734	10	985478	3.52E-07		intergenic region

	SNP2017367	10	1171376	8.59E-07		intergenic region
	SNP11993115	9	2352396	5.58E-07	RAPGEF5	Intron
	SNP2436003	11	1030333	1.02E-07	FDF5	intergenic region
Collagen	SNP6718977	22	6153896	1.11E-07	CAMTA1	Intron
	SNP6718989	22	6155091	4.12E-07		Intron
	SNP6718993	22	6155142	5.19E-07	GRM7	Intron
	SNP2787387	12	565792	5.88E-07	BLM	intergenic region
Water	SNP4515482	18	9571099	4.54E-07	WDR76	intergenic region
	SNP4909554	2	5891326	6.03E-07	EOMES	intergenic region
	SNP5834328	2	98086673	4.40E-07	RIMS2	Intron
	SNP5834329	2	98086675	4.57E-07		Intron
Protein	SNP5834331	2	98086679	4.96E-08		Intron
	SNP5834332	2	98086680	9.17E-07		Intron
	SNP7677715	3	1745186	7.00E-07	HNRNPU	intergenic region
	SNP9002473	34	730178	7.45E-07	SPTBN1	intergenic region

Table S2. Information of all significant CNV of all meat quality trait.

Phenotype	CNV_ID	CHR	BP	Fat	Location	Genes
	CNV2111	1	139615501	0.00045175	Intron	WASH7
Fat	CNV4246	32	792501	8.83E-05	intergenic region	TAP1/DMB
	CNV4493	4	39917001	0.00031535	Intron	GAB1
	CNV1695	1	29139501	9.97E-05	Intron	RB1
	CNV2235	1	182300001	0.00059693	Intron	SRPK2
	CNV2937	18	11891001	0.0008088	Intron	Mlxip
water	CNV4251	32	927001	0.00063141	intergenic region	Brd2
	CNV4256	32	974501	0.00031636	intergenic region	COL11A1/TAP1
	CNV4263	32	1031001	1.79E-05	intergenic region	C4/CENPA
	CNV4936	7	14985001	0.00028672	intergenic region	FAM178A
	CNV2720	15	14747001	0.00065294	intergenic region	DOCK2/FAM196B
protein	CNV2732	15	19055501	0.00022305	Intron	FBXO38
	CNV5086	8	21149001	0.00025544	intergenic region	ACBD6/XPR1
Collagen	CNV4562	4	63374001	0.00078465	intergenic region	CCSER1
